# The combination of paeonol, diosmetin-7-*O*-*β*-*D*-glucopyranoside, and 5-hydroxymethylfurfural from *Trichosanthis pericarpium* alleviates arachidonic acid-induced thrombosis in a zebrafish model

**DOI:** 10.3389/fphar.2024.1332468

**Published:** 2024-02-29

**Authors:** Shenghua Lin, Honglin Ma, Shanshan Zhang, Wei Fan, Chuanlin Shen, Jiayu Chen, Meng Jin, Kun Li, Qiuxia He

**Affiliations:** ^1^ Biology Institute, Qilu University of Technology (Shandong Academy of Sciences), Jinan, China; ^2^ Department of Gastroenterology, The First Affiliated Hospital of Shandong First Medical University and Shandong Provincial Qianfoshan Hospital, Jinan, China; ^3^ Science and Technology Service Platform, Qilu University of Technology (Shandong Academy of Sciences), Jinan, China

**Keywords:** *Trichosanthis pericarpium*, thrombosis, arachidonic acid, zebrafish, coagulation cascade, inflammation

## Abstract

*Trichosanthis fruit* (TF) is a classic medicinal material obtained from Shandong, China. The peel of this fruit (*Trichosanthis pericarpium*, TP) is known to exert anti-thrombotic effects. However, the anti-thrombotic active components and mechanisms of TP have yet to be fully elucidated. Combined with zebrafish models and high-performance liquid chromatography (HPLC), this study evaluated the endogenous anti-thrombotic effects with the combination of three compounds from TP. First, we used HPLC to investigate the components in the water extract of TP. Next, we used the zebrafish model to investigate the anti-thrombotic activity of the three compound combinations by evaluating a range of indicators. Finally, the expression of related genes was detected by real-time quantitative polymerase chain reaction (qPCR). HPLC detected a total of eight components in TP water extract, with high levels of paeonol (Pae), diosmetin-7-*O*-*β*-*D*-glucopyranoside (diosmetin-7-*O*-glucoside), and 5-hydroxymethylfurfural (5-HMF). The most significant anti-thrombotic activity was detected when the Pae: diosmetin-7-*O*-glucoside:5-HMF ratio was 4:3:3. qPCR analysis revealed that the abnormal expression levels of *f2*, *fga*, *fgb*, *vwf*, *ptgs1*, and *tbxas1* induced by arachidonic acid (AA) were improved. The combination of Pae, diosmetin-7-*O*-glucoside, and 5-HMF may alleviate AA-induced thrombosis by inhibiting the inflammatory reaction, coagulation cascade reaction, and arachidonic acid metabolism pathways.

## Graphical Abstract

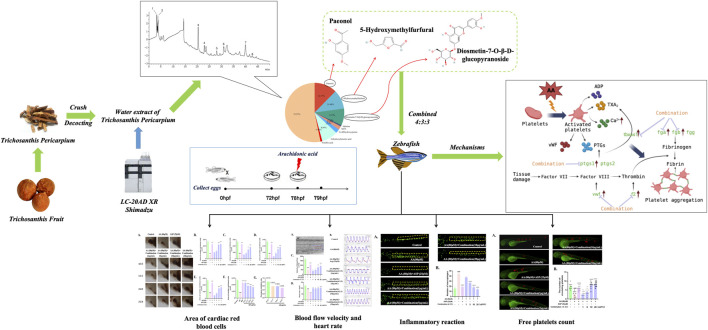

## 1 Introduction

Cardiovascular diseases (CVDs) have been listed by the World Health Organization as the most important threat to human life and health. Approximately 330 million individuals in China have been diagnosed with CVDs, and this number continues to increase (*Writing Committee of the Report on Cardiovascular Health and Diseases in China*, 2022). Thrombosis is one of the representative diseases of CVD and represents a serious threat to human health and safety (Stowell and Stowell, 2019; Chen et al., 2020), and venous thrombosis is an important factor that can lead to death worldwide (*The Lancet Haematology*, 2020). The mechanism responsible for thrombosis is the formation of blood clots in venous or arterial vessels, thereby limiting the blood flow (Ashorobi et al., 2023). A normal blood flow depends on a dynamic balance between plasma proteins, coagulation factors, inflammatory factors, cytokines, vascular endothelium, and other factors. If there is an imbalance in homeostasis, the probability of thrombosis will significantly increase (Ashorobi et al., 2023).

At present, there are two main options to treat thrombosis, namely, thrombolysis and anticoagulation, especially antiplatelet aggregation. Anticoagulants are predominantly used to inhibit the function of coagulation factors in the coagulation cascade reaction (Chapin and Hajjar, 2015; Pant et al., 2018). During antiplatelet aggregation, the platelet activation pathways are targeted by drugs, including platelet receptor pathways, thromboxane pathways, and protease-activated receptors (Depta and Bhatt, 2015). Aspirin (ASP) is a typical antiplatelet aggregation drug that inhibits platelet aggregation by the acetylation of cyclooxygenase (COX), thus inhibiting the conversion of arachidonic acid (AA) into prostaglandin G2 (PGG2), thereby reducing the production of thromboxane (Lichtenberger et al., 2017; Ornelas et al., 2017) while specifically eliminating AA.

Traditional Chinese medicine (TCM) is becoming increasingly accessible to a wider body of subjects, and it can be used to both prevent and treat thrombosis with a mild effect, few side effects, and high efficiency on multiple targets (Qu et al., 2019). *Trichosanthis fruit* (TF) is the mature and dry fruit of the Cucurbitaceae plants *Anguina kirilowii* Maxim. Kuntze the peel, seeds, and fruit from these plants can all be used as medicinal materials. TF can clear heat and eliminate phlegm (Qingre Titan), broaden the chest and disperse nodules (Kuanxiong Sanjie), and moisten dryness and smoothen the intestine (Runzao Huachang) (The Chinese Pharmacopoeia Commission, 2020). Kuanxiong Sanjie is a treatment method that uses prescriptions such as warming yang and dispersing cold, eliminating phlegm and turbidity, removing blood stasis and regulating qi, or other treatment procedures, and plays a useful role in dispersing obstruction, activating yang, and widening chest (Sanjie). Removing blood stasis and regulating qi is a common method that is used to treat thrombus in TCM.

Chinese researchers have reported that *Trichosanthis pericarpium* (TP) can protect against angina, hypoxia, ischemia-reperfusion damage, and other cardiovascular illnesses (Gao et al., 2015; Zhu et al., 2017; Zhang et al., 2018). Other studies have shown that TP can exert an anti-thrombotic effect. One previous study showed that the levels of endothelin, thromboxane B_2_ (TXB_2_), and myocardial malondialdehyde (MDA) were significantly reduced in the plasma of rats suffering from acute myocardial ischemia when injected with TP extract (Zhao et al., 2014). In addition, *in vitro* tests showed that AA-induced platelet aggregation and thromboxane A_2_ (TXA_2_) production were significantly inhibited following the injection of TP; these effects were dose-dependent (Ling et al., 1988). Yang et al. recorded electrocardiogram (ECG) and hemorheology parameters from 690 elderly patients with acute ischemic encephalopathy and found that the injection of TP led to a significant improvement in these two indicators (Yang et al., 2015). Furthermore, *in vitro* experiments, *in vivo* experiments in animal models, and clinical trials have proven that TP can exert anti-thrombotic activity, although the compounds responsible for the anti-thrombotic role of TP have yet to be elucidated. AA is the precursor of TXA_2_ in platelets, which is then converted into TXA_2_, a powerful inducer of platelet aggregation. Studies have shown that AA can directly activate platelets, induce platelet aggregation, and form thrombi. AA-induced thrombosis is thought to be representative of both venous and arterial thrombi (Jagadeeswaran et al., 2016; Zhu et al., 2022). Therefore, in the present study, we investigated the active components and mechanisms of TP using AA to generate a zebrafish model of thrombosis. Our aim was to provide new concepts and reference guidelines to investigate the anti-thrombotic activity and mechanisms of other medicinal herbs in the future.

## 2 Materials and methods

### 2.1 Medicinal materials and reagents

TF was purchased from Hebei Dazhong Pharmaceutical Co., Ltd., and it was produced in Shandong Province, China. The Chinese name for this fruit is Gualou, a mature and dry fruit of the Cucurbitaceae plant *Anguina kirilowii* (Maxim.) Kuntze. TF was cultivated in an environment with sufficient sunlight, good ventilation conditions, and no pollution. AA was purchased from Beijing Solarbio Science & Technology Co., Ltd. Aspirin (ASP) was purchased from AbMole BioScience Inc. Houston, United States of America. Paeonol (Pae), diosmetin-7-*O*-*β*-*D*-glucopyranoside (diosmetin-7-*O*-glucoside), and 5-hydroxymethylfurfural (5-HMF) were purchased from Sichuan Weikeqi Biological Technology Co., Ltd. Dimethyl sulfoxide (DMSO), acetic acid, and methanol were purchased from Sinopharm Group Chemical Reagent Co., Ltd. Phenylthiourea (PTU), tricaine, and o-dianisidine were purchased from Sigma, United States. Acetonitrile was purchased from Tedia Fairfield, OH, United States. The purity of these compounds is greater than 98%.

In this study, we utilized a range of instruments, including a SZX16 fluorescence microscope with a DP2-BSW image acquisition system (Olympus, Tokyo, Japan), a HPG-280BX light incubator (Harbin Donglian Electronic Technology Development Co., Ltd., China), a thermocycler (Model 9000; ABI, Waltham, MA, United States), a fluorescence quantitative thermocycler (LC96; Roche, South San Francisco, CA, United States), a high-performance liquid chromatography system (HPLC, LC-20AD XR, Shimadzu, Japan), and a BMG LABTECH microplate reader (SPECTROstar Nano, Germany).

### 2.2 Zebrafish

The zebrafish has become a highly respected animal model for rapid drug screening by virtue of a range of traits, including its short development cycle. The zebrafish strains used in this study were wild zebrafish AB and transgenic zebrafish *Tg* (coro1a: EGFP) and *Tg* (CD41: EGFP). Breeding was carried out in accordance with the published methods (Westerfield, 2007). Zebrafish embryos were collected and cultured in embryo-culture water E3 (5 mM NaCl, 0.17 mM KCl, 0.33 mM CaCl_2_, and 0.33 mM MgSO_4_•7H_2_O) and observed every day for embryonic development. Six hours after fertilization, we added 3% propylthiouracil (v/v) to the culture medium to prevent the zebrafish embryos from producing melanin. The Biology Institute of Shandong Academy of Sciences (3071027331728) granted permission for the use of zebrafish for research (approval code: SWS20220323; date of approval: 23 March 2022).

### 2.3 Composition analysis of the TP water extract

#### 2.3.1 Preparation of the TP water extract by decoction

First, we selected high-quality TF and divided it into peel and seeds. The peel was then ground into a powder. Then, 50 g of the peel powder was mixed with 500 mL of filtered water, heated, refluxed twice through a distillation unit (for 2 h each time), filtered, and combined with the filtrate. Then, the product was decompressed, concentrated, and freeze-dried to obtain a water extract of TP.

#### 2.3.2 Component content identification by HPLC

HPLC fingerprint analysis was performed using a SHIMADZU LC-20AD XR system (Japan) equipped with a COSMOSIL HILIC (hydrophilic interaction liquid chromatography) column (4.6 × 250 mm). Acetonitrile was used as solvent A, and a 0.2% acetic acid solution was used as solvent B. The following procedure was used for analysis: 0–7 min, 100A%→99A%; 7–10 min, 99A%→94A%; 10–15 min, 94A%→88A%; 15–45 min, 88A%→80A%; and 45–60 min, 80A%→50A%. The injection volume was 10 μL, and the flow rate was set to 1 mL/min. The column temperature was 30°C. Meanwhile, we analyzed the standard compounds under the same conditions, as mentioned above, and recorded their retention time. By comparing retention times, the components of the TP water extract were identified.

### 2.4 Experimental exposure of zebrafish

In order to investigate the anti-thrombotic active substances in TP, we next investigated the three major components of the TP extract that have been previously reported to have anti-inflammatory and anti-platelet aggregation properties or were previously detected in herbs and demonstrated to exhibit anti-thrombotic activity (Liu et al., 2004; Xu, 2011; Ye et al., 2016; Xu et al., 2021). We selected three specific compounds: paeonol (pae), diosmetin-7-*O-β-D*-glucopyranoside (diosmetin-7-*O*-glucoside), and 5-hydroxymethylfurfural (5-HMF).

When investigating the anti-thrombotic activity of the three compounds individually, we found that none of the compounds exhibited significant anti-thrombotic activity in zebrafish (Supplementary Figure S1). Thereafter, we investigated the anti-thrombotic effects of the original content ratio of the three compounds in TP in accordance with the peak area revealed by chromatography. The ratio of pae:diosmetin-7-*O*-glucoside:5-HMF was approximately 4:3:3 in the TP extract. Then, we specifically investigated the anti-thrombotic effects and compared other ratios of these three compounds to identify the best ratio.

We selected healthy zebrafish at 3 days post-fertilization (dpf) and transferred them into 24-well plates (5 fish per well). We set up a control group, an AA group, a 25-μM aspirin group, and a compound group (1, 5, 10, and 20 μg/mL). These substances were all mixed with DMSO as the solvent; the maximum proportion of DMSO in the exposure experiment was 5‰. Therefore, in order to eliminate the influence of the solvent on the experimental results, a 5‰ DMSO equilibrium experiment variable was added to the blank group. The culture plate was placed into the incubator at 28°C for 6 h. Then, we added AA to all the experimental groups except for the blank group until the final concentration was 80 μM (Ma et al., 2021; Zhu et al., 2022) and cultured for 1.5 h. In this experiment, each group involved three replicate wells, and each experiment was repeated three times.

### 2.5 *O*-dianisidine staining

After the zebrafish had been incubated with a combination of pae, diosmetin-7-*O*-glucoside, and 5-HMF, we then used o-dianisidine to stain the red blood cells in order to visualize thrombosis (Lin et al., 2023). First, we aspirated the culture media from the culture dish and added 1 mL of the o-dianisidine dye solution. Then, we incubated the solution for 15 min in the dark and used 100% DMSO to wash away the dye solution three times. Then, we randomly selected 10 zebrafish for stereoscopic microscopy and acquired relevant images. Red blood cells in the hearts of zebrafish were then analyzed (area and staining intensity) by Image-Pro Plus software.

### 2.6 Comparison of anti-thrombotic activity between the TP water extract and the combination of compounds (4:3:3)

To demonstrate the significant anti-thrombotic activity of the combination of compounds (4:3:3), we next investigated the anti-thrombotic activity of the water extract of TP by performing zebrafish exposure experiments, as described in Section 2.4. We used several concentrations of the TP water extract (5, 10, 20, and 40 μg/mL). At 20 μg/mL, the water extract of TP and the combination of compounds (4:3:3) were compared with regards to their anti-thrombotic activity. Then, we calculated the thrombus inhibition rate (TIR) of the water extract of TP and the combination of compounds (4:3:3) by applying an established formula (Li et al., 2023), as shown below.

$$\text{TIR}=\frac{S_{\text{sample}}-S_{\text{model}}}{S_{\text{control}}-S_{\text{model}}}.$$

### 2.7 Measurement of the blood flow velocity in zebrafish tails

We selected 10 zebrafish at random from each group. These fish were anesthetized until they had lost all movement and then transferred to a slide. Then, we used a stereo microscope (Olympus, Tokyo, Japan) fitted with a camera to record a video of the blood flow within the blood vessels of the tail of each fish (Parker et al., 2014). Each zebrafish was photographed for 10 s; then, we used Zebra Blood software (v1.3.2, ViewPoint, Lyon, France) to process the video and determine the blood flow velocity. First, we calibrated the blood vessel detection region to remove any interference from adjacent blood vessels when assessing the primary vessel. Then, the software program was used to detect the movement of red blood cells within the detection area, thus allowing the determination of blood flow velocity. This software detects changes in pixel density and combines these changes with the vessel diameter to generate a flow rate in nL/s for every frame (Parker et al., 2014; Zhu et al., 2020).

### 2.8 Measurement of the heart rate in experimental zebrafish

After the exposure experiment, we used the culture media to wash the zebrafish. Then, we waited for the zebrafish to stabilize on the slide before we used a counter to record the number of heart contractions for 15 s under a microscope. These data were then used to determine the heart rate in beats per minute.

### 2.9 The anti-thrombosis mechanisms associated with the combination of compounds

#### 2.9.1 The effect of combination on macrophage response

Using the AA-induced zebrafish model of thrombosis, we selected a macrophage-specific fluorescent *Tg* (coo1a:EGFP) zebrafish strain for the quantification of inflammation. Following exposure, the zebrafish were washed with the culture media and MS-222 was added for anesthesia. From each group, we randomly chose 10 zebrafish for examination and image acquisition with an Axio Zoom V16 body-type green fluorescent stereoscopic microscope (Zeiss, Germany). To evaluate inflammation in zebrafish, we performed macrophage counts on the trunk of each zebrafish.

#### 2.9.2 Quantification of circulating platelets in zebrafish

Next, we selected a platelet fluorescent *Tg* (CD41:eGFP) zebrafish strain to investigate platelet circulation. Zebrafish were treated with a combination of pae, diosmetin-7-*O*-glucoside, and 5-HMF, washed with culture media, and anesthetized. Then, 10 zebrafish were selected from each group for examination and image acquisition with a SZX16 fluorescence microscope (Olympus, Tokyo, Japan) and a DP2-BSW image acquisition system. The number of circulating platelets in the peripheral blood vessels of each zebrafish was quantified manually.

#### 2.9.3 Determination of the levels of TXA_2_ and Ca^2+^ in zebrafish

We used a TXA_2_ ELISA Kit (Shanghai Lengton Bioscience Co., Ltd.), in accordance with the manufacturer’s instructions, to detect the concentration of TXA_2_ in experimental zebrafish (Fang et al., 2014 ; Fei et al., 2017). In brief, 30 zebrafish were collected from each group after exposure. Each fish from each group was first weighed and lysed to obtain a supernatant. Then, we recorded the optical density (OD) at 450 nm and generated a standard curve using the method described in the Supplementary Material.

In addition, we used a Calcium Colorimetric Assay Kit to detect the levels of Ca^2+^ in each zebrafish (Cheng et al., 2020; Li et al., 2020). In total, 30 zebrafish were collected from each group after exposure. Each fish from each group was first weighed and lysed to obtain a supernatant. Then, we recorded the optical density (OD) at 575 nm and generated a standard curve using the method described in the Supplementary Material.

#### 2.9.4 Analysis of gene expression levels

Total RNA was extracted from 30 zebrafish with a Total RNA Isolation Kit. Then, we used a HiScript Ⅱ Reverse Transcriptase Kit to convert mRNA to single-strand cDNA. Finally, RT-qPCR was performed using the ChamQ Universal SYBR qPCR Master Mix two-step quantitative RT-qPCR technique. All three kits were purchased from Vazyme Biotechnology Co., Ltd. *β-actin* was used as an internal reference gene. Following RT-qPCR, the CT value for each group was determined with LightCycler 96 SW 1.1 software. The 2^−ΔΔCT^ method was used to determine the relative gene expression (Lin et al., 2023). The sequences of the primers used in this part of the study are shown in Supplementary Table S1.

### 2.10 Statistical analysis

All experiments were replicated three times, and all data are given as mean ± standard error of the mean (SEM). Data analysis was performed with GraphPad Prism version 9.0 software. One-way analysis of variance (ANOVA) (Bartlett’s test) was used to identify significant differences between each group and the AA group; *p* < 0.05 indicated a significant difference.

## 3 Results

### 3.1 Analysis of the chemical constituents of the TP water extract by HPLC

**FIGURE 1:**
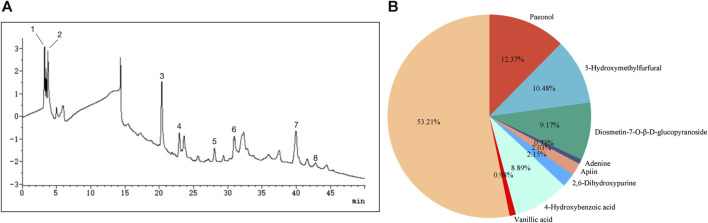
Analysis of chemical constituents. **(A)** Ultraviolet chromatogram of the water extract of TP-260 nm. (1) paeonol, (2) 5-hydroxymethylfurfural, (3) diosmetin-7-O-β-D-glucopyranoside, (4) adenine, (5) apiin, (6) 2,6-dihydroxypurine, (7) 4-hydroxybenzoic acid, and (8) vanillic acid. **(B)** Pie chart showing the relative proportions of the eight chemical components.

Eight compounds were identified in the water extract of TP by HPLC at the UV wavelength of 260 nm (Figure 1A). The eight compounds were paeonol, 5-hydroxymethylfurfural, diosmetin-7-*O-β-D*-glucopyranoside, adenine, apiin, 2,6-dihydroxypurine, 4-hydroxybenzoic acid, and vanillic acid. Figure 1B shows the ratio of the relative content of each compound. Table 1 provides comprehensive details relating to the molecular weight, relative content, and formula of these eight compounds. The top three compounds, in terms of relative content, were paeonol, diosmetin-7-*O-β-D*-glucopyranoside, and 5-hydroxymethylfurfural (at a ratio of approximately 4:3:3).

**TABLE 1:** Chemical composition of the water extract of TP, as determined by HPLC.

NO.	Name	Retention time/min	Formula	Molecular weight	Peak area	Relative content (%)
1	Paeonol	3.263	C_9_H_10_O_3_	116.174	57,665	12.38
2	5-Hydroxymethylfurfural	3.749	C_6_H_6_O_3_	126.11	48,872	10.49
3	Diosmetin-7-O-β-D-glucopyranoside	20.368	C_22_H_22_O_11_	462.409	42,788	9.18
4	Adenine	23.312	C_5_H_5_N_5_	135.13	3,386	0.73
5	Apiin	28.062	C_26_H_28_O	564.49	9,475	2.03
6	2,6-Dihydroxypurine	30.903	C_5_H_4_N_4_O_2_	152.111	10,036	2.15
7	4-Hydroxybenzoic acid	39.915	C_7_H_6_O_3_	138.13	41,619	8.90
8	Vanillic acid	42.814	C_8_H_8_O_4_	168.15	4,550	0.98

### 3.2 Four different combinations of the three compounds inhibited thrombosis in zebrafish

**FIGURE 2:**
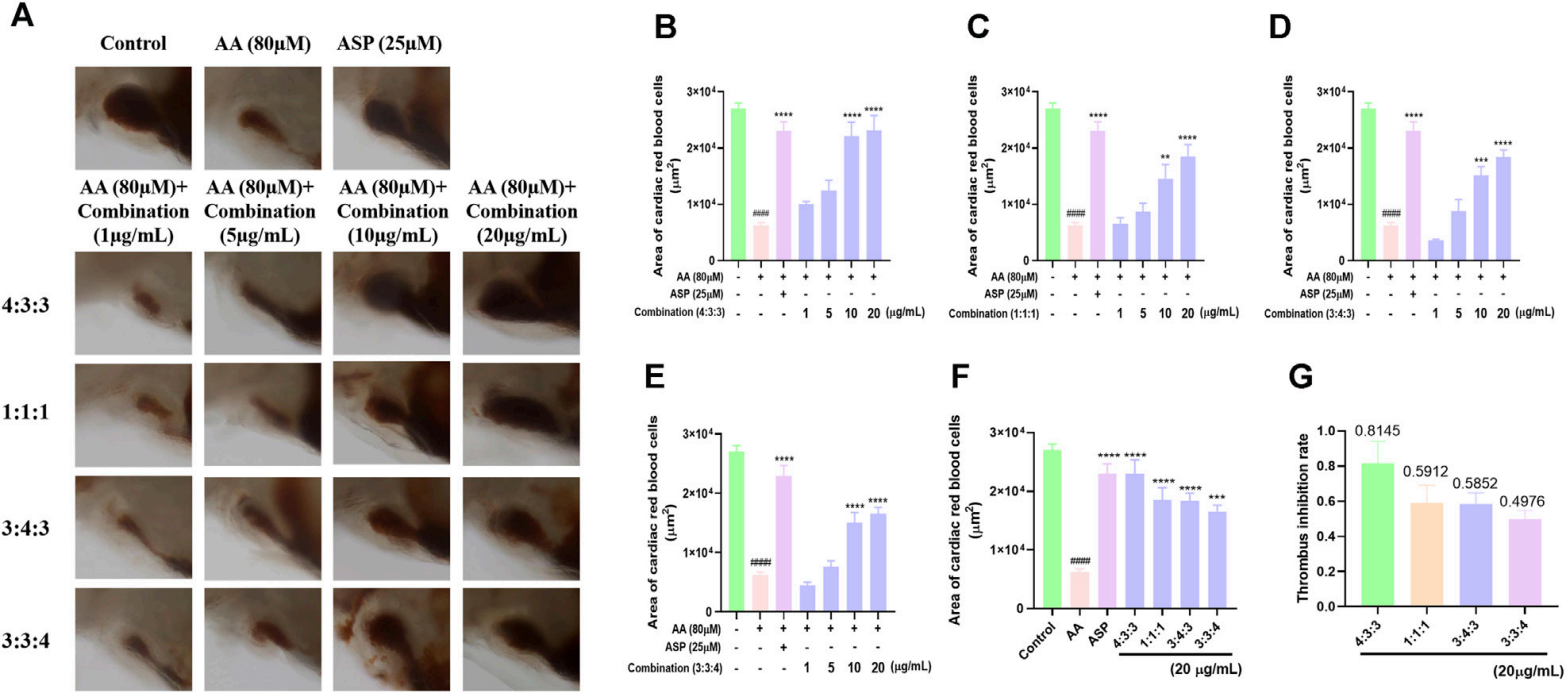
Effects of different ratios of the combination of compounds on thrombosis in zebrafish. **(A)** Morphological effects of the four different ratios of the three components of TP on AA-induced thrombosis in zebrafish (n = 10); **(B–E)** histogram showing the statistical effect of the four different ratios of the three components of TP on the area of AA-induced erythrocytes in the thrombosed hearts of zebrafish (n = 10); **(F)** comparison of the effects of the four different ratios at the same concentration (20 μg/mL) on AA-induced thrombosis in zebrafish (n = 10); **(G)** statistical analysis of the thrombus inhibition rate of different combinations (4:3:3; 1:1:1; 3:4:3; 3:3:4) (n = 10).^####^ *p* < 0.0001 vs. control, ^****^ *p* < 0.0001, ^***^ *p* < 0.001, and ^**^ *p* < 0.01 vs. AA.

Following AA treatment, the blood volume returning to the hearts of experimental zebrafish decreased significantly (Figure 2A). This finding showed that AA induced thrombosis in the blood vessels. When the four combinations were added respectively, we found that the blood volume flowing back to the heart increased significantly (Figure 2A). This indicated that the four combinations alleviated AA-induced thrombosis in zebrafish. As the concentration of the combinations increased, the area of red blood cells flowing back to the heart of the zebrafish also increased significantly; this occurred in a concentration-dependent manner (Figure 2B–E). Finally, we compared the area of red blood cells flowing back to the heart and the thrombus inhibition rate at a concentration of 20 μg/mL. These results showed that a 4:3:3 ratio of pae:diosmetin-7-*O*-glucoside:5-HMF exhibited the optimal anti-thrombosis effect induced by AA among all experimental groupings (Figures 2F and G). Therefore, we used a 4:3:3 ratio in all subsequent experiments.

### 3.3 The combination of compounds exhibited a more significant inhibitory effect on thrombosis in zebrafish

**FIGURE 3:**
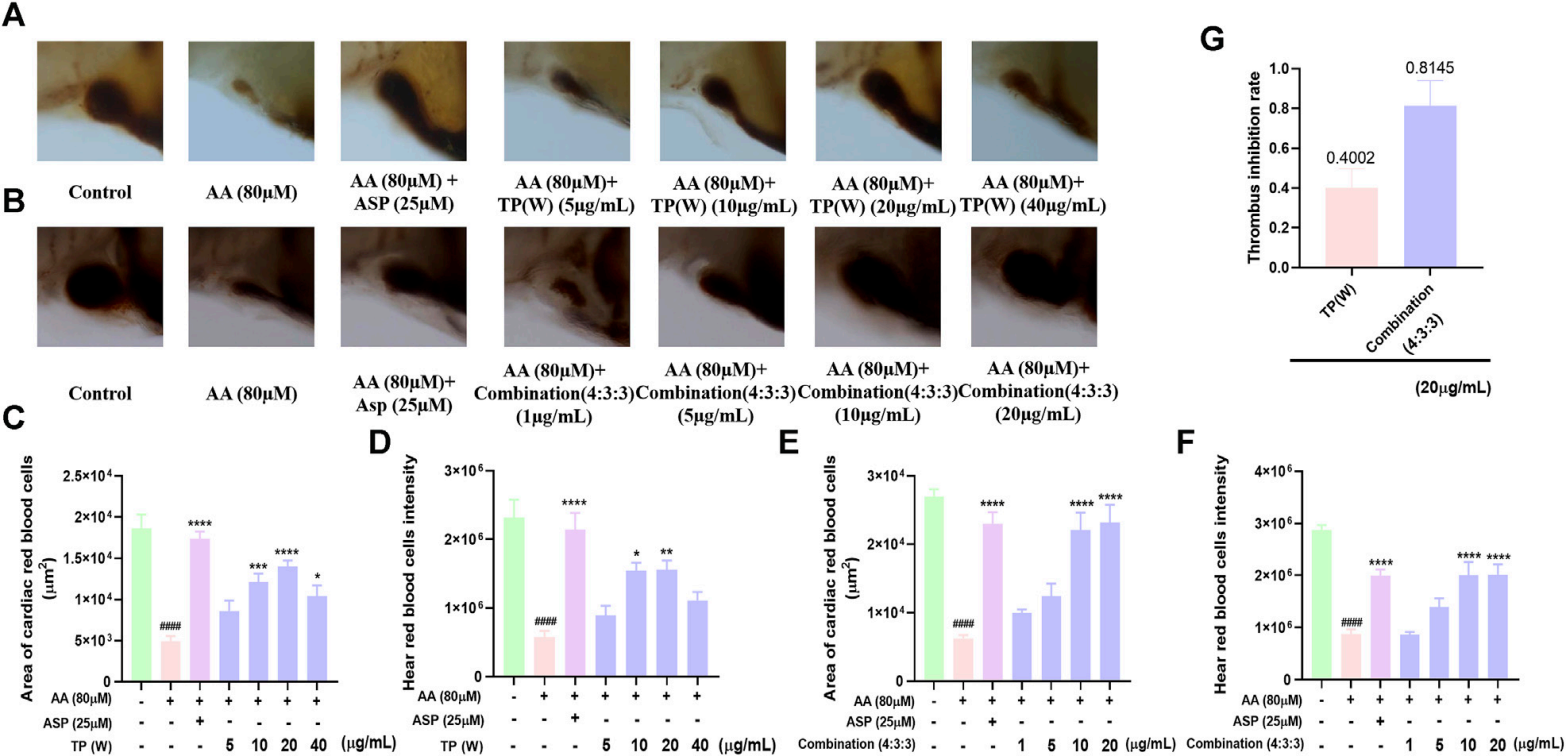
Comparison of the anti-thrombotic activity of a water extract of TP in a 4:3:3 combination. **(A)** Characterization of the anti-thrombotic activity of a water extract of TP (n = 10); **(B)** characterization of anti-thrombotic activity of the 4:3:3 combination (n = 10); **(C, D)** statistical analysis of the staining area and staining intensity of cardiac red blood cells in AA-induced zebrafish thrombi by a water extract of TP (n = 10); **(E, F)** statistical analysis of the staining area and staining intensity of cardiac red blood cells in AA-induced zebrafish thrombi by the 4:3:3 combination (4:3:3) (n = 10); **(G)** statistical analysis of the thrombus inhibition rate by a water extract of TP and the effects of different combinations (4:3:3) (n = 10). ^####^ *p* < 0.0001 vs. control, ^****^ *p* < 0.0001,^***^ *p* < 0.001, ^**^ *p* < 0.01, and ^*^ *p* < 0.05 vs. AA.

Next, we investigated the anti-thrombotic activity of the TP water extract and found that the anti-thrombotic activity of the water extract of TP was most significant at a concentration of 20 μg/mL (Figures 3C,D). However, we found that both the staining area of cardiac red blood cells and the staining intensity decreased at 40 μg/mL. This may be due to the increase in the concentration of the TP water extract, which shows a certain degree of toxicity, but the toxicity is weaker than the drug at this time, so it still has an anti-thrombotic function at this concentration. On the other hand, we think that the concentration of other inactive compounds in the TP water extract has reached a certain point that restricts the effectiveness of the active substances. As a result, the anti-thrombotic activity is comparatively weaker at this concentration. To compare the anti-thrombotic activity of the TP water extract in a 4:3:3 ratio, we next determined the thrombus inhibition rate. Analysis showed that the thrombus inhibition rate of the combination at a ratio of 4:3:3 was twice that of the TP water extract. This indicated that the 4:3:3 combination exhibited the best anti-thrombotic activity.

### 3.4 Combination treatment improved the blood flow velocity and heart rate

**FIGURE 4:**
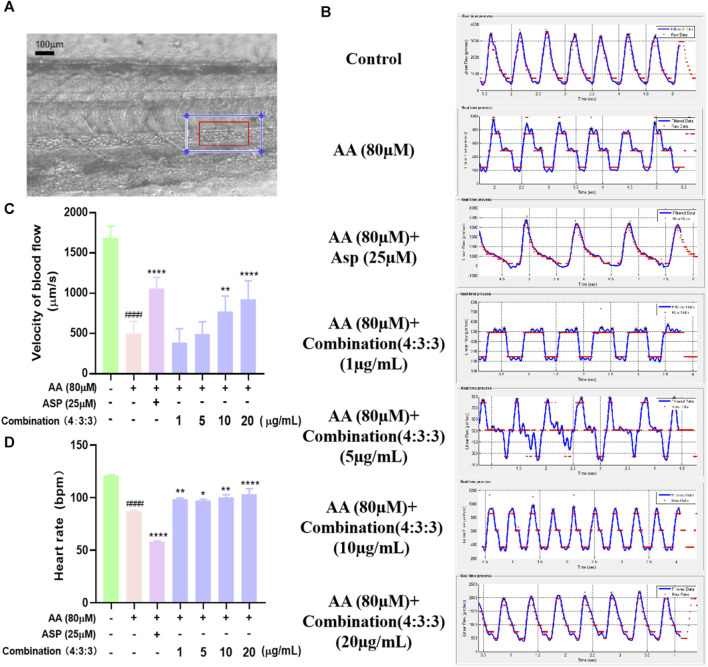
Effects of the 4:3:3 combination on the blood flow velocity and heart rate in zebrafish. **(A)** Arterial vascular diagram of a zebrafish tail. **(B, C)** Changes in the blood flow velocity in the tail of zebrafish with increasing concentrations of the 4:3:3 combination (n = 10). **(D)** Changes in the heart rate of zebrafish (n = 10). ^####^ *p* < 0.0001 vs. control, ^****^ *p* < 0.0001, ^**^ *p* < 0.01, and ^*^ *p* < 0.05 vs. AA.

Prior to processing videos with Zebra Blood software, it was necessary to perform calibration; the change in the frame rate following calibration was then used to quantify the rate of blood flow velocity (Figure 4A). As shown in Figures 4B and C, after AA treatment, the blood flow velocity in the tails of zebrafish decreased significantly; this showed that AA-induced thrombosis limited the flow velocity of blood flow. However, the 4:3:3 combination significantly increased the blood flow velocity in zebrafish, thus indicating that the 4:3:3 combination alleviated AA-induced thrombosis.

Next, we determined the heart rate of zebrafish at 3dpf and found that the AA-induced heart rate was reduced in zebrafish when treated with the 4:3:3 combination and that all concentrations alleviated the reduction in the heart rate in zebrafish. The most pronounced heart rate inhibitory effect in zebrafish was observed when the combination concentration reached a concentration of 20 μg/mL.

### 3.5 Anti-inflammatory effects of the combination treatment

**FIGURE 5:**
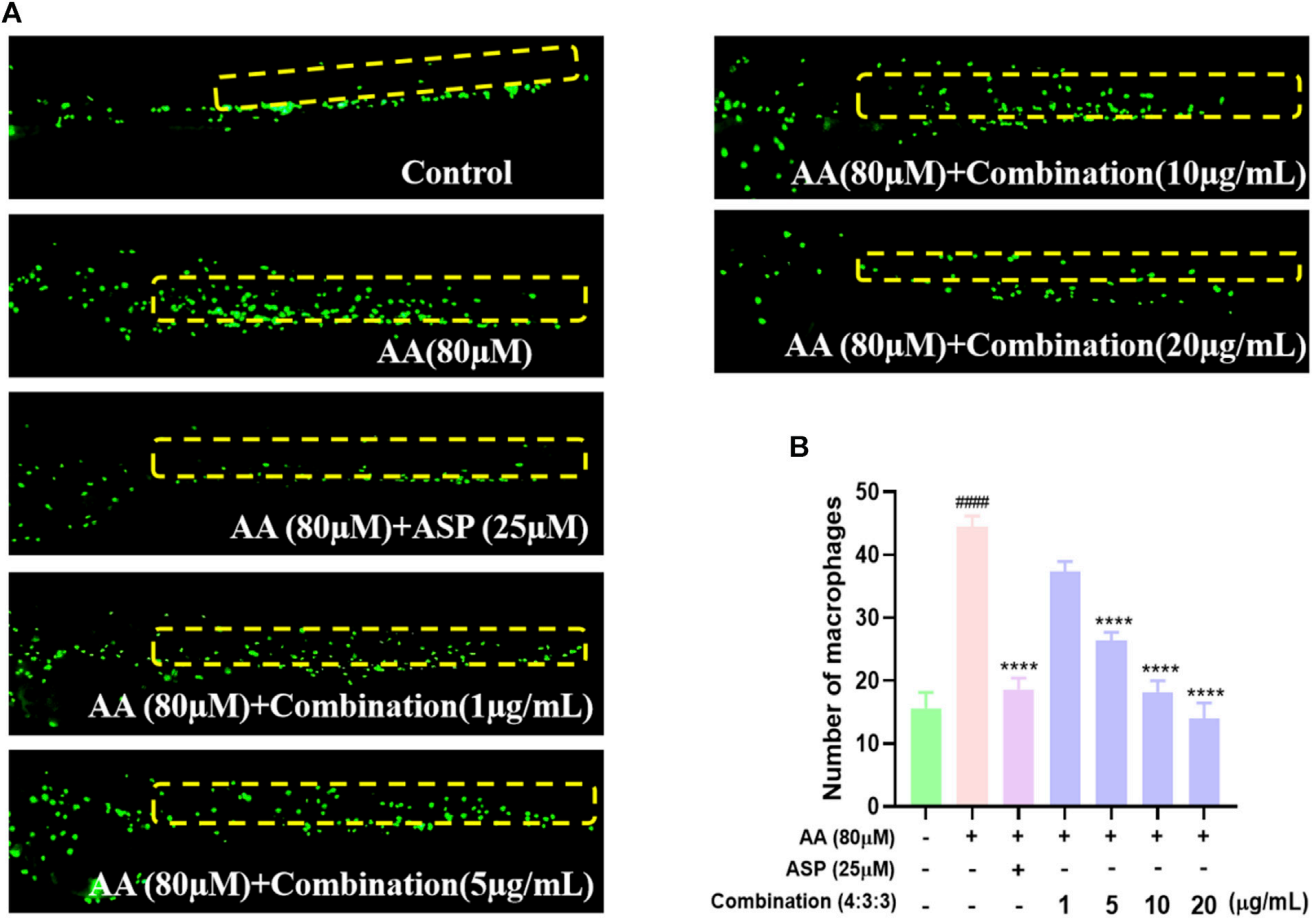
Effect of the 4:3:3 combination on inflammation in zebrafish. **(A)** Migration and aggregation of macrophages in the trunk of zebrafish (n = 10). **(B)** Number of macrophages in the trunk of zebrafish (n = 10). ^####^ *p* < 0.0001 vs control; ^****^ *p* < 0.0001 vs AA.

The migration and aggregation of macrophages represent direct indications of the inflammatory status of zebrafish. The tail region of the AA group exhibited the highest number of macrophage aggregates. ASP is known to exert anti-inflammatory properties; thus, the tail regions of zebrafish in the ASP group exhibited fewer macrophage aggregates (Figure 5A, B). In addition, incubation with the 4:3:3 combination reduced the number of macrophages aggregated in the tail of zebrafish; this effect was concentration-dependent (Figure 5A, B).

### 3.6 Combination treatment improved the number of platelets in the peripheral blood circulation of zebrafish

**FIGURE 6:**
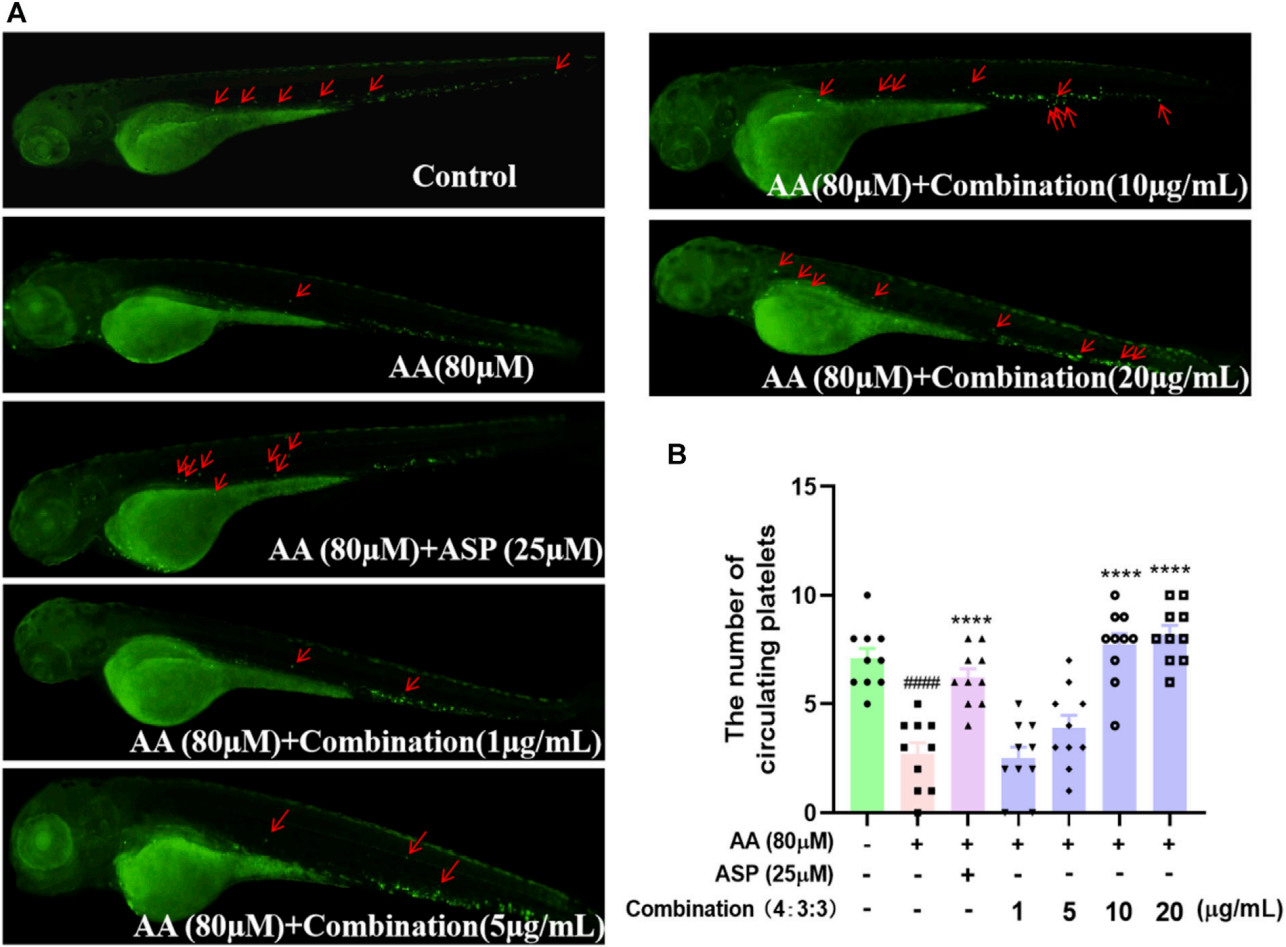
Effect of the 4:3:3 combination on circulating platelets in the peripheral vessels of zebrafish. **(A)** Morphology of circulating platelet distribution in zebrafish (n = 10). **(B)** Number of platelets circulating in each group of zebrafish (n = 10). ^####^ *p* < 0.0001 vs control; ^****^ *p* < 0.0001 vs. AA.

Next, we used a *Tg* (CD41:eGFP) strain of zebrafish to investigate the circulating platelets. We found that zebrafish in the AA group had the lowest number of circulating platelets (Figure 6) and that the circulating platelets moved more slowly *in vivo* than those in the control group, thus suggesting that circulating platelets were heavily depleted by the thrombi in zebrafish of the AA group; furthermore, platelet circulation was reduced due to the formation of thrombi. As the 4:3:3 concentration of the combination increased, the number of circulating platelets gradually increased, and the platelets moved faster when compared to that of the fish in the AA group (Figure 6). In other words, the number of circulating platelets in zebrafish that were consumed by thrombi decreased. This finding indicated that the 4:3:3 combination inhibited AA-induced thrombosis, thus causing the circulation velocity of platelets to increase and the quantity of consumed circulating platelets to decrease.

### 3.7 Ca^2+^ levels and qPCR detection results

**FIGURE 7:**
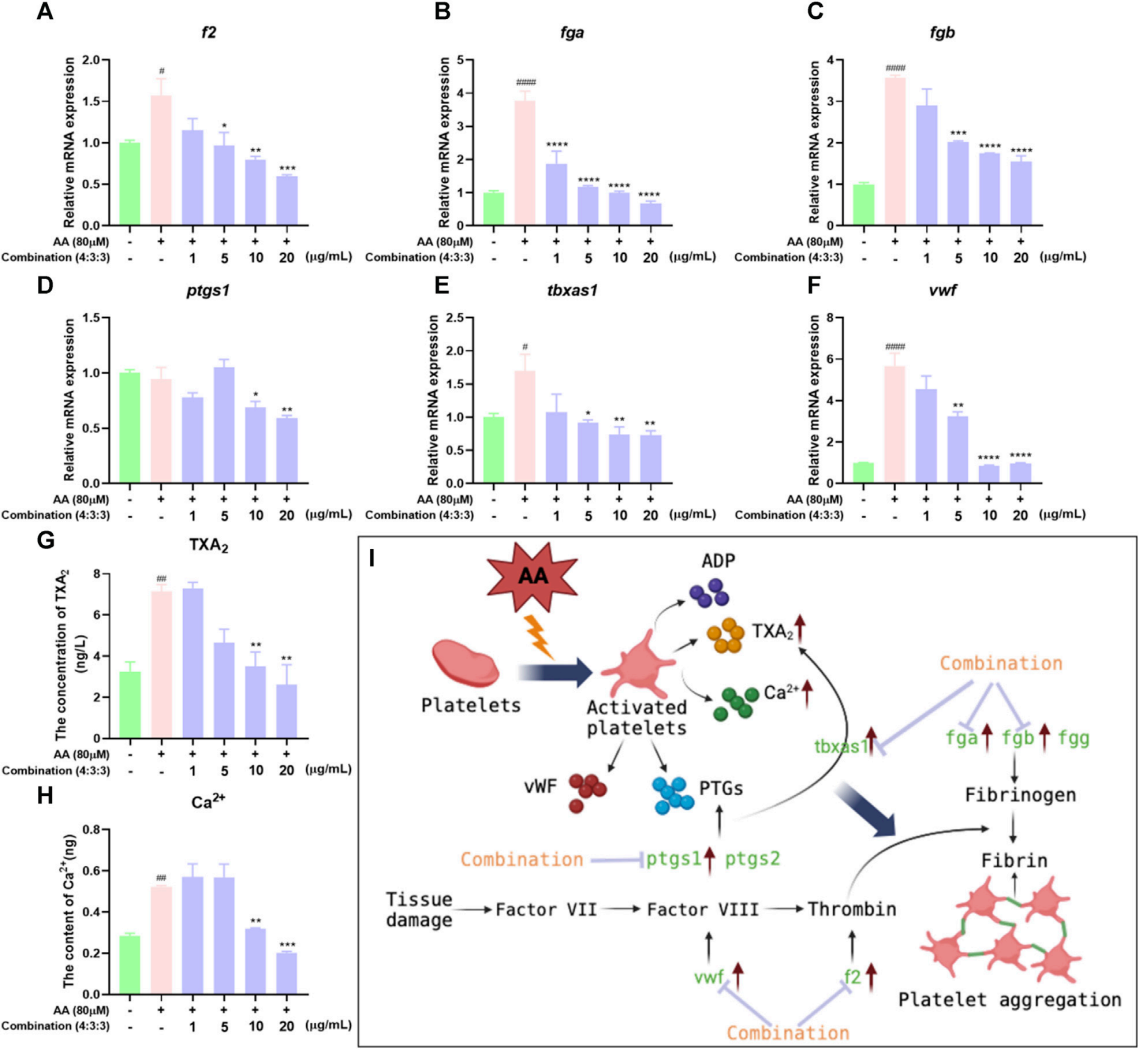
Effects of the 4:3:3 combination on the levels of TXA_2_ and Ca^2+^ and gene expression levels in zebrafish. **(A–F)** Changes in the expression levels of genes in zebrafish when treated with the 4:3:3 combination (n = 3). **(G, H)** Changes in the levels of TXA_2_ and Ca^2+^ in zebrafish when treated with the 4:3:3 combination (n = 5). **(I)** Diagram showing the proposed mechanism for the 4:3:3 combination (4:3:3) when treating AA-induced thrombosis. ^####^ *p* < 0.0001, ^#^ *p* < 0.05 vs. control, ^****^ *p* < 0.0001, ^***^ *p* < 0.001, ^**^ *p* < 0.01, and ^*^ *p* < 0.05 vs. AA.

The process of thrombosis can depend heavily on platelet activity and the coagulation cascade (Sheng et al., 2020). First, we measured the levels of Ca^2+^ in zebrafish. The levels of Ca^2+^ in zebrafish progressively decreased with increasing concentrations of the 4:3:3 combination (Figure 7G). Next, we investigated changes in the expression levels of genes relating to coagulation and the key factors that activate platelets by qPCR. We found that *f2*, *fga*, *fgb*, and *vwf* exhibited reduced levels of expression with increasing concentrations of the 4:3:3 combination (Figure 7A–C, F). The expression of the *ptgs1* gene was downregulated relative to the AA group at the combination concentrations of 10 μg/mL and 20 μg/mL (Figure 7D). The expression of the *tbxas1* gene was decreased with increasing concentrations of the 4:3:3 combination (Figure 7E). Finally, we constructed a potential mechanistic diagram of the 4:3:3 combination for the treatment of AA-induced thrombosis (Figure 7H).

### 3.8 Combination treatment inhibited the expression of inflammatory genes

**FIGURE 8:**
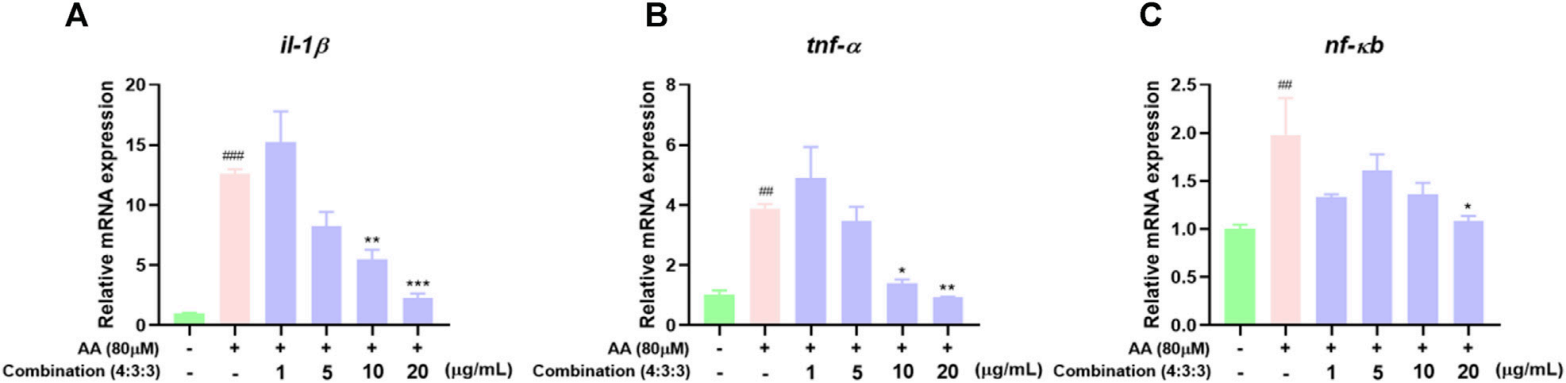
Effects of the 4:3:3 combination on the expression levels of inflammatory-related genes in zebrafish (*n* = 3). **(A–C)** Changes in the expression levels of genes (il-1β, tnf-α, and nf-кb) in zebrafish when treated with the 4:3:3 combination (*n* = 3). ^###^ *p* < 0.001, ^##^ *p* < 0.01 vs control, ^***^ *p* < 0.001, ^**^ *p* < 0.01, and ^*^ *p* < 0.05 vs. AA.

In previous experiments, we found that the 4:3:3 combination exerted anti-inflammatory effects. Therefore, we detected the expression levels of pro-inflammatory genes by qPCR. These results showed that AA can upregulate the expression levels of *il-1β*, *tnf-α*, and *nf-кb* genes. The expression levels of these genes were significantly downregulated after incubation with the 4:3:3 combination (Figure 8A–C). This showed that the 4:3:3 combination may play a role in inflammatory pathways, such as the TNF pathway, to alleviate AA-induced thrombosis.

## 4 Discussion

Vascular endothelial cell damage, along with alterations in the blood flow velocity and the coagulation system, can all trigger thrombosis (Cotter and Pillai, 2019). Therefore, the mechanism of thrombosis is complex. However, the science underlying TCM follows the principle of systematic treatment, where the combination of multiple drugs can work together to achieve better therapeutic effects. An increasing body of evidence now indicates that multiple components in TCM can act on multiple targets and regulate various biological processes, thereby exerting a range of pharmacological effects (Xu et al., 2021). The multi-component effects of TCM can also exert various mechanisms of action, including anti-inflammatory and antioxidant effects, thereby improving the treatment efficacy and reducing adverse reactions in patients (Wang et al., 2018). However, multi-components can also generate limitations where other components of TCM can affect the active components, at least to a certain extent. Therefore, it is of great significance to perform research relating to the discovery, enrichment, mechanisms of action, and even the ratio of multiple components in the active ingredients of TCM. In the present study, we investigated the anti-thrombotic active components of TP, as well as their combined anti-thrombotic activities and mechanisms of action.

Based on the HPLC results, we found that the levels of pae, diosmetin-7-*O*-glucoside, and 5-HMF were relatively high; many previous studies have demonstrated that these three compounds can exert anti-thrombotic activities (Liu et al., 2004; Ye et al., 2016; Xu et al., 2021). Initially, we investigated the individual anti-thrombotic activities of these three compounds using the zebrafish model. These three compounds did not exhibit significant anti-thrombotic activity below a concentration of 20 μg/mL. Inspired by the theory of TCM compatibility, we combined these three compounds and found that their combination could exert a significant anti-thrombotic effect. Therefore, we investigated the anti-thrombotic activity and mechanisms of action of their combination.

The zebrafish model has a wide range of applications in thrombosis research (Gibbins and Mahaut-Smith, 2012); this is because the platelet function in zebrafish shares many common features with humans, including GPIIb-IIIa, the arachidonic acid metabolism pathway, and numerous coagulation factors (Jagadeeswaran et al., 1999; Lang et al., 2010). Therefore, it is meaningful to investigate the anti-thrombotic activity of the combination using the zebrafish model. Then, we selected AA to generate a zebrafish model of thrombus. The induction of zebrafish thrombosis by AA is caused by the production of TXA_2_ by AA under the action of TXA_2_ synthase. This disrupts the balance of PGI_2_/TXA_2_, causing platelet aggregation and vasoconstriction, ultimately leading to thrombosis (Patrono et al., 1984; Bijak and Saluk-Bijak, 2017). The length of thrombi in the tail vein of zebrafish has been reported to be positively proportional to the cardiac erythrocyte area (Qi et al., 2017). Therefore, we selected the erythrocyte region in zebrafish hearts as an indicator of thrombus formation. When the zebrafish were treated with AA, there was a significant reduction in the erythrocyte area in the heart, thus indicating that the zebrafish model of thrombosis had been generated successfully. In addition, measuring blood flow velocity is also considered a very effective tool for determining the severity of thrombosis (Parker et al., 2014). In damaged vessels, platelets are activated to form a thrombus; this causes a reduction in blood flow velocity *via* a complex mechanism of interactions between multiple receptors and ligands (Estevez and Du, 2017; Yeung et al., 2018). Here, we demonstrated that the 4:3:3 combination exerted an obvious anti-thrombotic activity. Compared with the activity of the TP water extract, the 4:3:3 combination exhibited a TIR that was twice that of the TP water extract. In addition, when measuring the blood flow velocity of zebrafish, we found that both ASP and the 4:3:3 combination could inhibit thrombosis and improve the velocity of blood flow. However, we also found that the distance between the two peaks of a blood flow fluctuation map in the ASP group was larger than that in the 4:3:3 combination group. This finding indicated that ASP reduced the heart rate of zebrafish. This could result in a side effect in patients who suffer from both thrombi and heart disease, such as an insufficient blood supply to the heart and brain. However, the 4:3:3 combination was able to stabilize the heart rate, and in this regard, the 4:3:3 combination had significant advantages over ASP.

Inflammatory reactions are inevitable during thrombosis; this represents a protective response produced by the body and aims to clear the damaged and infected pathogens and promote tissue repair. However, when the inflammatory response is excessive or persistent, a large number of inflammatory factors will be released, leading to abnormal endothelial cell function, thus promoting platelet activation and endothelial cell damage, ultimately promoting thrombosis (Nayak and Vedantham, 2012; Schroer et al., 2023). Therefore, eliminating inflammatory reactions is meaningful for the treatment of thrombosis (Singh and Masuda, 2005). Previous clinical studies have confirmed a significant increase in the serum levels of inflammatory factors during venous thrombosis. TNF-α is the earliest inflammatory factor that responds during the inflammatory response period, and it can directly participate in the inflammatory response. During this response, a large number of inflammatory cells aggregate, thus triggering the activation of many cells, including lymphocytes and macrophages (Henke et al., 2006), and promoting the release of interleukins, such as IL-1β and IL-10, which act as inflammatory factors (Satoh et al., 2015). IL-10 can bind to the specific receptor CD210, which is distributed on peripheral blood mononuclear cells (PBMCs), thus activating tyrosine kinase and Jak kinase, while also activating transcription factors and signal transduction to inhibit the activation of NF-кB (Mercurio and Manning, 1999; Sun and Chen, 2022). Therefore, the high expression levels of TNF-α, IL, and NF-кB are closely related to vascular injury. In the AA-induced zebrafish model of thrombus, we found that a large number of macrophages accumulated in the trunk, thus indicating the occurrence of an inflammatory response. However, the number of macrophages decreased gradually in a dose-dependent manner when treated with the 4:3:3 combination. Finally, we determined the expression levels of the *tnf-α*, *il-1β*, and *nfкb* genes in zebrafish by qPCR (Figure 8). The trends in the expression levels of these genes were consistent with the reduced number of macrophages. This suggested that the potential anti-thrombotic mechanism responsible for the effects of the 4:3:3 combination was related to the regulation of the *tnf-α*, *il-1β*, and *nfкb* gene expression.

Platelets and coagulation factors play an important role in the process of thrombosis. Normal endothelial cells prevent thrombosis by releasing anticoagulants and inhibiting platelet-activating factors (Mason et al., 1977; Mehta and Malik, 2006). When the endothelial cells incur damage, platelets are activated and release a large number of active factors, such as ADP, TXA_2_, and Ca^2+^, which subsequently lead to platelet aggregation (Warner et al., 2011; Wijeyeratne and Heptinstall, 2011). A series of coagulation reactions can induce thrombosis *via* the coagulation cascade (Zhou et al., 2017). The earliest reaction involves the von Willebrand factor (vWF), an essential mucopolysaccharide protein that is released by leukocytes into the bloodstream and binds to GPIb protein receptors on platelets to form loose emboli when blood vessels are damaged, ultimately leading to thrombosis (Gragnano et al., 2017; Shahidi, 2017). Moreover, the *fga*, *fgb*, and *fgg* genes encode for fibrinogen and play key roles in the coagulation cascade (Vo et al., 2013). Fibrinogen can specifically bind to platelet glycoprotein IIb-IIIa to cause platelet aggregation (Farrell et al., 1992; Ma et al., 2021). Furthermore, the levels of fibrinogen (Fga, Fgb, and Fgg) and thrombin (F2) increase, thus inducing hypercoagulability and thrombosis (Chopard et al., 2020). Here, we demonstrated that the 4:3:3 combination could inhibit thrombosis and reduce the consumption of circulating platelets (Figure 6). Moreover, the qPCR results showed that the 4:3:3 combination significantly increased the gene expression levels of the platelet-activating factor (*vwf*) and various other factors involved in the coagulation cascade (*fga*, *fgb*, and *f2*). These data suggested that the 4:3:3 combination may have inhibited thrombosis by inhibiting the coagulation cascade and the activation of platelets.

Prostaglandin–endoperoxide synthase (PTGS and COX) and TXA_2_ synthetases are key proteins in the early stages of the AA pathway. PTGSs are a group of enzymes that can convert AA into eicosanoids, which are active metabolites and include prostaglandins and thromboxanes (Sheng et al., 2020). PTGS has two isoforms, PTGS1 (COX1) and PTGS2 (COX2), of which the more dominant form is PTGS1 (Grosser et al., 2002). Research has shown that low-dose APS can permanently acetylate PTGS1, prevent AA from being converted, and inhibit thrombosis (Baigent and Patrono, 2003). Moreover, PTGS1 converts AA into PGG2, which is chemically unstable and can be hydrolyzed to form prostaglandin H2 (PGH2), which can be subsequently converted to TXA_2_, a platelet agonist and vasoconstrictor, *via* the action of the TXA_2_ synthase (Smith and Malkowski, 2019; Badimon et al., 2021). These two steps in the AA metabolic pathway represent crucial hubs for thrombosis. Nevertheless, in our model of thrombus, AA had a significant effect on the expression levels of *tbxas1* but had no significant effect on the expression levels of *ptgs1*. However, we consider that AA may control the protein activity of PTGS1 at the post-transcriptional level, although this hypothesis requires further investigation. In the present study, we investigated the expression levels of *ptgs1* and *tbxas1* by qPCR and found that their expression levels could be inhibited by a higher concentration of the 4:3:3 combination. Based on these results, we believe that the 4:3:3 combination may reduce the levels of TXA_2_ by inhibiting the expression levels of *ptgs1* and *tbxas1*, alleviating the balance of PGI_2_/TXA_2_, inhibiting platelet activation and the coagulation cascade, and, thus, inhibiting thrombosis. Therefore, the anti-thrombotic effects of the 4:3:3 combination may also be closely related to the AA metabolic pathway.

## 5 Conclusion

TF, as a medicinal and edible plant, plays an important role in preventing CVDs. In this study, we investigated the anti-thrombotic activity and potential mechanisms of action of a combination of three components from TP. Our analysis demonstrated that the combination may inhibit thrombosis by exerting effects on the coagulation cascade reaction, inflammatory reaction pathway, and the arachidonic acid metabolism pathways. Future research should investigate the combined anti-thrombotic effects of the two compounds in combination. This would not only reduce costs in the subsequent pharmaceutical process but would also provide reference guidelines for investigating the efficacy of other TCMs in the future.

## Data Availability

The data presented in the study are deposited in the Figshare repository, accession number 10.6084/m9.figshare.25241974.
