# Supplementary material for: The combination of paeonol, diosmetin-7-O-β-D-glucopyranoside, and 5-hydroxymethylfurfural from Trichosanthis pericarpium alleviates arachidonic acid-induced thrombosis in a zebrafish model
Source: Front Pharmacol. 2024 Feb 29;15:1332468. doi: 10.3389/fphar.2024.1332468 (PMC10937350; doi:10.3389/fphar.2024.1332468)
Supplement: Supplementary file 1 [file Table1.DOCX]

Supplementary material

# None of the three compounds had anti-thrombotic activity

In order to find the anti-thrombotic active components in TP, we explored the alone anti-thrombotic effect of Pae, diosmetin-7-*O*-glucoside and 5-HMF in TP. The results show that none of the three compounds had anti-thrombotic activity (figure S1 A-D).


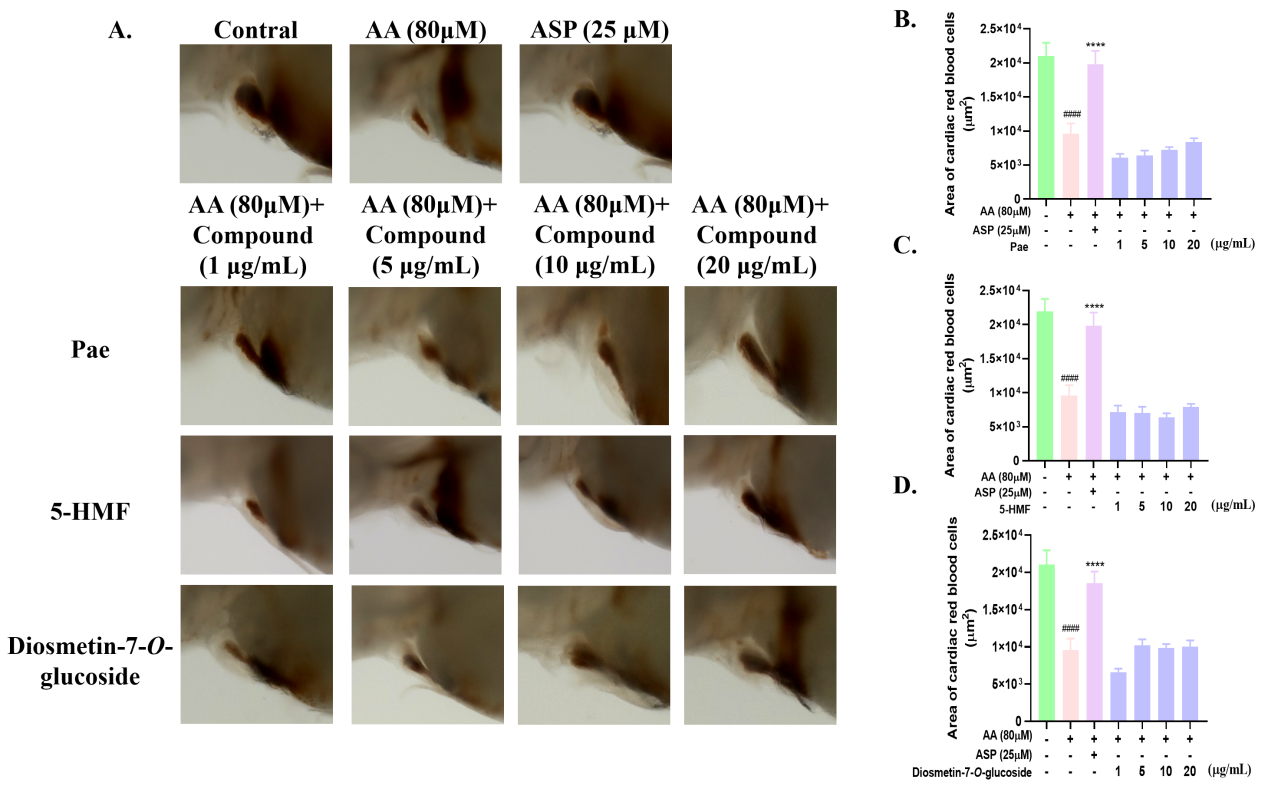


**Supplementary Figure 1.** Effect of Pae, diosmetin-7-*O*-glucoside and 5-HMF on the thrombosis of zebrafish larvae. A. Morphology of the effect of Pae, diosmetin-7-*O*-glucoside and 5-HMF on AA-induced thrombosis in zebrafish (n=10). B-D. Histogram of the statistical effect of Pae, diosmetin-7-*O*-glucoside and 5-HMF on the area of AA-induced erythrocytes in thrombosed hearts of zebrafish (n=10). ^####^*P*<0.0001 vs control, ^****^*P*<0.0001 vs model.

**2 The information of primer sequence**

**Supplementary table 1.**

| Genes | Forward (5’-3’) | Reverse (5’-3’) |
| --- | --- | --- |
| *β-actin* | GCATTGCTGACCGTATGCAC | ACTCCTGCTTGCTGATCCAC |
| *il-1β* | TCCAAACGGATACGACCAGC | TGCGAATCTTCATACGCGGT |
| *nf-*к*b* | AGGCCAAAGACACTGTTCGG | GGAAAGGTTGTGGGGTCCAT |
| *tnf-α* | GGAGAGTTGCCTTTACCGCT | CCTGGGTCTTATGGAGCGTG |
| *fga* | TTTTTGGGGATGACCTCGGG | TTGAACATCCCGCTCTGACC |
| *fgb* | TCAGAGAGCCAAGTGCCAAG | ACATCTGCGAGTCCTCTCCT |
| *vwf* | GTTCCTGCTGAGAGCGTCTT | ACGACCGATGTTTGTGCTCT |
| *f2* | TCATCGTCCGCCTTGGAAAA | TCGGTTCAGGTTTTCCTTCCA |
| *ptgs1* | CTGGTGAAGAAAGCCCGACT | ATAGTAGCCCGTTCTGGTGC |
| *tbxas1* | AGACAAGCAGCAACACGCTA | GGCATAATCCACCGTCTCGT |

**3 Measurement of TXA_2_ and Ca^2+^levels in zebrafish larvae**

We prepare standard solution with concentrations of 800, 400, 200, 100, 50 and 0 ng/L, respectively. Then, the OD value of the standard solution was measured using a microplate reader (SPECTROstar Nano, Germany). OD value measured by the standard solution was used to fit the logistic curve (four parameters) in the ELISA scale software. The concentration of TXA_2_ was the abscissa, and the OD value was the ordinate. Finally, we used the curve to calculate the concentration of TXA_2_ in each group of zebrafish larvae.

We prepare standard solution with concentrations of 1.0, 0.8, 0.6, 0.4, 0.2, 0.1 and 0 mM, respectively. Then, the OD value of the standard solution was measured using a microplate reader (SPECTROstar Nano, Germany). OD value measured with the standard solution was used to fit the linear equation curve in the ELISA scale software. Ca^2+^ content were the abscissa and OD value was the ordinate. Finally, we used the curve to calculate the Ca^2+^ content of each group of zebrafish larvae.

The standard curves measured first are:

Y(TXA_2_)=(2.9867+0.01678)/[1+(x/29.36401)^1.06012^]-0.01678, r^2^=0.99911 Y(Ca^2+^)=0.34604+1.13908x, r^2^=0.99853
